# Molecular epidemiology identifies the expansion of the DENV2 epidemic lineage from the French Caribbean Islands to French Guiana and mainland France, 2023 to 2024

**DOI:** 10.2807/1560-7917.ES.2024.29.13.2400123

**Published:** 2024-03-28

**Authors:** Raphaëlle Klitting, Géraldine Piorkowski, Dominique Rousset, André Cabié, Etienne Frumence, Alisé Lagrave, Anne Lavergne, Antoine Enfissi, George Dos Santos, Laurence Fagour, Raymond Césaire, Marie-Christine Jaffar-Bandjee, Nicolas Traversier, Patrick Gérardin, Rayane Amaral, Lucie Fournier, Lucie Leon, Frédérique Dorléans, Muriel Vincent, arbovirus genomics diagnostic laboratories working group, Albin Fontaine, Anna-Bella Failloux, Nazli Ayhan, Laura Pezzi, Gilda Grard, Guillaume André Durand, Xavier de Lamballerie, Arbovirus genomics diagnostic laboratories working group members, Laura Verdurme, Marie Hervo, Alexia Barbry, Pierre Bancons, Valérie Suez-Panama, Isabelle Komla-Soukha, Jean-François Carod, Magalie Demar, Didier Musso, Alain Berlioz-Arthaud, Sandra Fabienne, Kate Lebouteiller, Esther Christine, Michèle Adélaïde, Fabrice Ghisalberti, Anne Ligneureux, Céline Ben Cimon, Mahery Ramiandrisoa, Geoffrey Masson, Paul Séraphin

**Affiliations:** 1 National Reference Center for Arboviruses, Inserm-IRBA, Marseille, France; 2 Unité des Virus Émergents (UVE: Aix-Marseille Univ, Università di Corsica, IRD 190, Inserm 1207, IRBA), Marseille, France; 3 Associated National Reference Center for Arboviruses, Virology unit, Institut Pasteur in French Guiana, Cayenne, French Guiana; 4 Service de Maladies infectieuses et tropicales, CHU de Martinique, Fort-de-France, France; 5 PCCEI, Université de Montpellier, INSERM, EFS, Montpellier, France; 6 CIC Antilles Guyane, INSERM CIC1424, Fort-de-France, France; 7 Associated National Reference Center for Arboviruses, CHU de la Réunion-Site Nord, Saint-Denis, Réunion, France; 8 Laboratoire de microbiologie, CHU de la Réunion-Site Nord, Saint-Denis, Réunion, France; 9 Laboratoire de virologie, CHU de Martinique, Fort-de-France, France; 10 Pôle de biologie territoriale, CHU de Guadeloupe, Pointe-à-Pitre, France; 11 CIC 1410, CHU Réunion, Saint-Pierre, France; 12 Santé publique France, Saint-Maurice, France; 13 Santé publique France, Cellule Antilles, Saint-Maurice, France; 14 Santé publique France - La Réunion, Saint-Denis, La Réunion, France; 15 The members of this group are listed under collaborators and at the end of the article; 16 Institut de Recherche Biomédicale des Armées (IRBA), Unité de virologie, Marseille, France; 17 Department of Virology, Arboviruses and Insect Vectors, Institut Pasteur, Paris, France

**Keywords:** arbovirus, dengue, surveillance, emergence, molecular epidemiology

## Abstract

In 2023, dengue virus serotype 2 (DENV2) affected most French overseas territories. In the French Caribbean Islands, viral circulation continues with > 30,000 suspected infections by March 2024. Genome sequence analysis reveals that the epidemic lineage in the French Caribbean islands has also become established in French Guiana but not Réunion. It has moreover seeded autochthonous circulation events in mainland France. To guide prevention of further inter-territorial spread and DENV introduction in non-endemic settings, continued molecular surveillance and mosquito control are essential.

In the region of the Americas, the year 2023 set an unwelcome record in terms of dengue virus (DENV) circulation, with more than 4.1 million suspected infections distributed across 42 countries and territories [1]. Regarding the French Caribbean Islands, where dengue surveillance is conducted year-round, epidemics of DENV serotype 2 (DENV2) were declared in Guadeloupe and Martinique simultaneously on 17 August 2023. Eight and 10 weeks later, the islands of Saint-Barthélemy and Saint-Martin respectively entered the epidemic phase for dengue, which was also caused by DENV2. Up to March 2024, over 30,000 suspected cases of DENV infection have been reported across all four islands [2]. In 2023, DENV2 was also active in other French overseas territories including French Guiana and the island of Réunion, both of which share strong ties with the French Caribbean Islands [3,4]. In this work, we provide evidence supporting that a single epidemic lineage circulating in Guadeloupe, Martinique, Saint-Barthélemy and Saint-Martin has become established in French Guiana but not in Réunion. Moreover, the epidemic strain appears to have caused autochthonous clusters of cases in mainland France.

## Description of the centres collecting and analysing genomic data

To describe virus circulation in the French Caribbean islands and identify potential exchanges between these islands, French Guiana and Réunion, the French National Reference Center for arboviruses in mainland France and associated centres in the French Caribbean Islands–Guiana region and in the Indian Ocean region generated and analysed virus genome data across these territories. Moreover, as nine clusters of autochthonous DENV cases were reported in mainland France in 2023 [5], sequence data from two DENV2 clusters were generated to investigate potential links with DENV2 circulating in the French Caribbean islands.

## Finding of a single DENV2 lineage in the French Caribbean Islands with repeated viral strain exchanges between islands

DENV2 sequences derived from cases identified in the French Caribbean Islands between February 2023 and January 2024 were obtained through convenience sampling, as described in the Supplementary Material. We generated and analysed 103 nearly complete DENV2 genome sequences (> 95% coding sequences (CDS)), 46 of which originated from infections in Guadeloupe, 51 from Martinique, three from Saint Barthélemy, one from Saint Martin, and two from French Caribbean Islands (exact location unknown); more information can be found in Supplementary Table 1. By inferring the phylogenetic relationships between these viral genomic sequences and a set of reference sequences representative of DENV2 genotypes, we found that all French Caribbean Island sequences belong to the cosmopolitan genotype.

**Figure 1:**
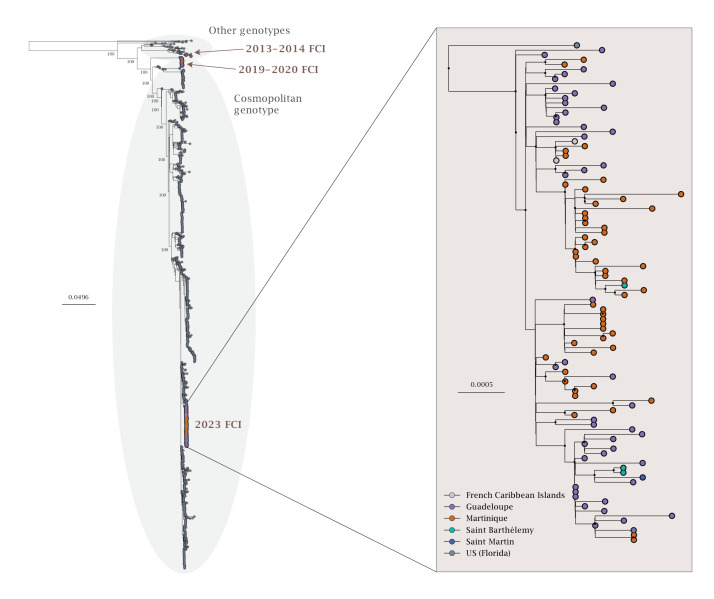
Phylogeny of the cosmopolitan genotype of DENV2 with a focus on sequences from the French Caribbean Islands epidemic, 2023–2024 (n = 1,348 sequences)

CDS: coding sequence; DENV2: dengue virus serotype 2; FCI: French Caribbean Islands.

To detail virus circulation between individual islands, we inferred a phylogeny based on a dataset combining all 103 virus genomes from the epidemic with all nearly complete genomes from the cosmopolitan genotype available in GenBank up to 22 November 2023 (Supplementary Table 1). In the resulting phylogenetic tree (Figure 1), French Caribbean Islands’ sequences from 2023 to 2024 form a monophyletic clade (bootstrap > 95), indicating that one main lineage is responsible for the current epidemic. Within this epidemic clade, sequences from Martinique and Guadeloupe are interspersed, suggesting that virus circulation involves exchanges between the two islands. Also, sequences from Saint Barthélemy are separated in different subgroups of French Caribbean sequences, suggesting that there have been multiple introductions from Martinique and/or Guadeloupe into this island. The only sequence obtained from Saint Martin Island also groups within the 2023–2024 French Caribbean clade, indicating that the epidemic lineage is also present on the island. 

Maximum-likelihood phylogeny of all genomes from the FCI epidemic combined with all DENV2 sequences from the cosmopolitan genotype covering more than 85% of the CDS available on GenBank up to 22 November 2023 and a set of reference sequences from other genotypes. Phylogenetic inference was performed using IQTREE2 under ModelFinder with ultrafast bootstrap approximation (1,000 replicates) [19,20]. The tree was rooted using the sylvatic genotype of DENV2. All sequences are coloured according to their geographic origin (for imported cases, the geographic origin corresponds to the source of importation identified via epidemiological surveillance, as described in Supplementary Table 1). In the main tree, bootstraps are shown for all nodes with more than 800 descendants and sequences from previous DENV2 circulation events in the French Caribbean islands are shown with arrows. In the French Caribbean Islands subtree, all nodes with a bootstrap support above 95 are highlighted with a black circle. The scale represents phylogenetic divergence, in nucleotide substitutions per site.

## The epidemic lineage emerged in 2022, following a new introduction into the French Caribbean Islands

To determine whether the epidemic clade stemmed from a new introduction or was circulating locally before the upsurge in cases, we also included in the phylogeny sequences from archived samples corresponding to infections identified during previous epidemics in the French Caribbean Islands (Supplementary Table 1). We observed that DENV2 genomes from the 2023 epidemic group with sequences from the United States (Florida 2023) and India (2021–2022) with good statistical support (bootstrap > 95) rather than with French Caribbean sequences from previous years (Figure 1). In particular, they are distinct from DENV2 cosmopolitan sequences from Martinique and Guadeloupe from the years 2019 and 2020. This topology suggests that virus circulation was most likely caused by a new introduction into the French Caribbean Islands.

To date the origin of the epidemic, we selected a subset of 75 DENV2 genomes and used Bayesian inference to evaluate the time of emergence of the epidemic clade. Using the best fitted model (Supplementary Table 2), we found that the time to the most recent common ancestor of the epidemic clade dates back to September 2022 (95% highest posterior density (HPD) interval: 24 June to 26 December 2022), an estimate that aligns with the timing of the resurgence of dengue cases that was observed in Guadeloupe starting in October 2022 [6], which preluded the epidemic.

## French Caribbean Island lineage strains found in French Guiana and autochthonous clusters in mainland France but not Réunion

**Figure 2:**
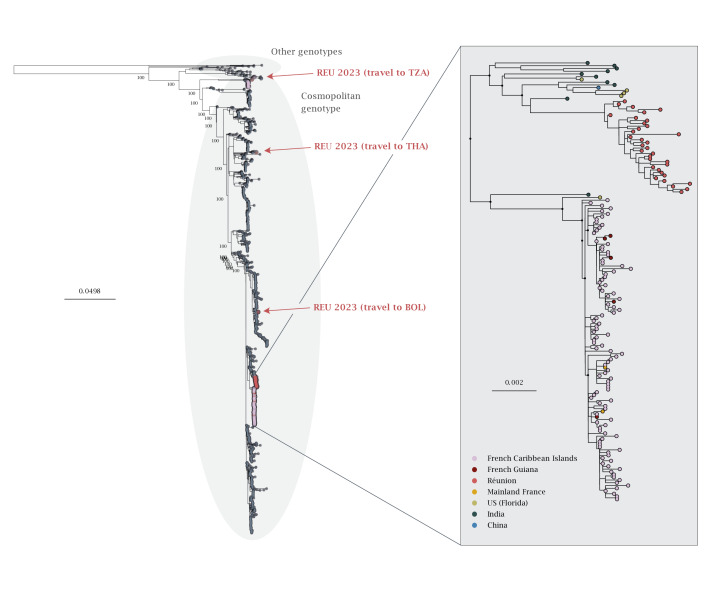
Phylogeny of the cosmopolitan genotype of DENV2 with a focus on sequences from French overseas territories, 2023–2024 (n = 1,394)

CDS: coding sequence; DENV2: dengue virus serotype 2; ISO: country International Organization for Standardization 3 code.

In 2023, DENV2 was active in other French overseas territories. In French Guiana, an epidemic of DENV is ongoing since July 2023. While DENV3 was the predominant serotype (> 95% of serotyped cases) until September 2023 [7], DENV2 increased in prevalence, initially in the ‘Ile de Cayenne’ district [8,9], and is now present across most districts and on par with DENV3 at the level of the territory [10]. In Réunion, DENV was circulating at an endemic level in 2023 (218 cases) [11], with detections of both DENV1 and DENV2, and infections reported throughout the year. Also, mainland France recorded six clusters of autochthonous DENV2 circulation starting in July 2023 [5], with two DENV2 sequences available, one for the cluster in Limeil-Brévannes (3 cases, Île-de-France region [12]) and one for the Gardanne cluster (4 cases, Provence-Alpes-Côte d'Azur region). To determine whether the virus lineages circulating in other French territories were related to the French Caribbean Islands epidemic, we conducted a new phylogenetic analysis (Figure 2) including – in addition to the original dataset – five DENV2 genomes from cases acquired in French Guiana, 42 DENV2 genomes from cases identified in Réunion, and two DENV2 sequences from autochthonous circulation events in mainland France. The additional sequences were selected according to convenience sampling, and information about them can be found in Supplementary Table 3. We found that all Guianese sequences group within the French Caribbean Islands clade. This result, combined with serotype prevalence data, indicates that the French Caribbean Island epidemic lineage is now circulating in French Guiana. In contrast, sequences from Réunion cluster separately from the French Caribbean Islands’ clade, suggesting that the viral strains circulating in Réunion are due to an introduction from a distinct location. Finally, the two sequences from autochthonous DENV2 circulation events in Limeil-Brévannes and Gardanne are part of the French Caribbean Islands’ clade, which indicates that these two events likely result from introductions from the Caribbean.

Maximum-likelihood phylogeny of all genomes from the French Caribbean Islands epidemic combined with sequences, from French Guiana, from Réunion island, from mainland France, and with all DENV2 sequences from the cosmopolitan genotype covering more than 85% of the CDS available on GenBank up to 22 November 2023. Phylogenetic inference was performed using IQTREE2 under ModelFinder with ultrafast bootstrap approximation (1,000 replicates) [19,20]. The tree was rooted using the sylvatic genotype of DENV2. All sequences are coloured according to their geographic origin (for imported cases, the geographic origin corresponds to the source of importation). In the main tree, bootstrap values are shown for all nodes with more than 800 descendants and sequences from Réunion islands (2023) not present in the subtree are shown with arrows (with the ISO code 3 of the of travel location when applicable). In the subtree, all nodes with more than 35 leaves and with a bootstrap support above 95 are highlighted with a black circle. The scale represents phylogenetic divergence, in nucleotide substitutions per site.

## Discussion

Dengue is endemo-epidemic in the French Caribbean Islands where it has caused several epidemics over the past decades including in 2013–2014 and in 2019–2021, during which distinct serotypes circulated on the islands of Guadeloupe and Martinique [13,14]. In 2013–2014, DENV serotype 4 (DENV4) dominated in Guadeloupe and DENV2 was the main serotype in Martinique, while in 2019–2021, DENV2 was dominant in Guadeloupe and DENV serotype 3 (DENV3) was the main serotype in Martinique. Here, we identify circulation dynamics which differ markedly from those past epidemics, with one main lineage present both in Guadeloupe and Martinique and a phylogenetic tree topology suggestive of exchanges between both islands. The successful spread of DENV2 on the island Guadeloupe only 2 years following a previous DENV2 epidemic is unexpected considering the broad and recent homotypic immunity in the island population, and in a context where other serotypes are actively circulating in Central America (including in French Guiana), notably DENV3 [1]. The past and current DENV2 lineages being quite distant phylogenetically, potential antigenic differences might explain the temporal proximity between the past and current circulation events. Other explanations may however apply, including a particularly high transmission efficiency of the current epidemic lineage.

In this work, we show that the epidemic clade stems from a new introduction in the French Caribbean Islands, but locating the source of this introduction is challenging in part because of limited genomic sampling around the French Caribbean Islands. We find that the closest relative to strains of the French Caribbean Islands epidemic clade is a strain from Florida collected in July 2023, which itself appears to be closely related to strains sampled in India in 2021. While the closest phylogenetic relative of the French Caribbean Islands epidemic clade strains appears to have been sampled in Florida, this location is not a likely geographic origin of the epidemic. Local DENV case numbers are usually limited in Florida [15], especially compared with other countries neighbouring the French Caribbean Islands, such as Honduras, Panama, and the Dominican Republic, which reported DENV2 circulation over recent years (2021–2022) according to the Pan American Health Organization (PAHO) [16,17]. The source of the epidemic affecting the French Caribbean Islands is likely located elsewhere, possibly in Central America, from where it may have also caused one – or more – local circulation events in Florida.

Our study presents several limitations. Here, we used near-complete (> 95%) CDSs to infer phylogenetic relationships among DENV genomes. The absence of up to 5% of the CDS and of the 3’ and 5’ untranslated regions (UTRs) of the genome may result in a loss of resolution in the tree topology [18]. This is nevertheless expected to have minimal impact on the parts of the tree with strong statistical support and thus on the conclusions of the study. Moreover, the limited number of sequences available for French Guiana may limit our ability to infer with certainty the directionality of the DENV2 lineage circulation between the French Caribbean islands and French Guiana. Our results are however, also supported by serotype prevalence data that indicate that DENV2 prevalence was extremely low in French Guiana before October 2023, making an importation from a location with high DENV2 case numbers to a location with low DENV2 case numbers much more likely than the opposite. Finally, our analysis of autochthonous DENV2 transmission events in Limeil-Brévannes and in Gardanne relies on a single sequence for each of the clusters, as the remainder of the cases were either negative by PCR or exhibited viral loads incompatible with sequencing. The three cases from Limeil-Brévannes belonged to the same family [12] and all four cases from Gardanne were located within a 1 km radius. Given the size of the clusters and the spatio-temporal vicinity among the few cases involved, it is likely that for each of these two clusters, un-sequenced cases share viruses of the same phylogenetic lineage as the sequenced case. In addition, as the French Caribbean islands constituted − by far − the most frequent source of imported cases in mainland France in 2023, it is also the most likely source of autochthonous circulation events independent of any sequence data analysis [5].

### Conclusion

In this work, we use virus genomic data to detail circulation dynamics during the French Caribbean Islands epidemic. We identify a change in local circulation trends compared with previous epidemics in the French Caribbean Islands and show that the lineage linked to this epidemic appears to have become established in French Guiana amidst an epidemic of DENV3. Finally, we provide evidence suggesting that strains belonging to the French Caribbean Island epidemic lineage also fuelled autochthonous DENV2 circulation events in mainland France, with at least two local DENV2 clusters involved. These results highlight the strong epidemiological connections between French territories and call for increased efforts in genetic monitoring to gain additional insights into those inter-territorial exchange dynamics and inform public health strategies, such as immunisation strategies, blood donor screening, information to travellers, or vector surveillance and control.

## Arbovirus genomics diagnostic laboratories working group members’ affiliations

Laboratoire Cerba, Frépillon, France: Laura Verdurme; Infectious Diseases Department, Eurofins-Biomnis Lyon, France: Marie Hervo, Alexia Barbry; Cerballiance Martinique: Pierre Bancons; Centre de ressource biologique de la Martinique, CHU de Martinique, France: Valérie Suez-Panama, Isabelle Komla-Soukha; Centre Hospitalier de l'Ouest Guyanais, Saint-Laurent-du-Maroni, French Guiana: Jean-François Carod; Laboratoire, Centre Hospitalier de Cayenne, Cayenne, French Guiana: Pr Magalie Demar; Laboratoires Eurofins Guyane: Didier Musso et Alain Berlioz-Arthaud; Laboratoires Eurofins Martinique: Sandra Fabienne, Kate Lebouteiller, Esther Christine et Michèle Adélaïde, Fabrice Ghisalberti; Centre Hospitalier Ouest Réunion: Anne Ligneureux; Inovie Laboratory (Réunion): Céline Ben Cimon; Cerballiance (Réunion): Mahery Ramiandrisoa; Medical biology laboratory of Saint-Benoît (Réunion): Geoffrey Masson; Bioaustral Laboratory (Réunion): Paul Séraphin.

## Supplementary Data

304000 Supplementary Material
