# Authors’ correction for Euro Surveill. 2024;29(13)

**DOI:** 10.2807/1560-7917.ES.2024.29.25.240620c

**Published:** 2024-06-20

**Authors:** Eurosurveillance editorial team

**Affiliations:** 1 European Centre for Disease Prevention and Control (ECDC), Stockholm, Sweden

In the article entitled ‘Molecular epidemiology identifies the expansion of the DENV2 epidemic lineage from the French Caribbean Islands to French Guiana and mainland France, 2023 to 2024’ by Klitting et al., published on 28 March 2024, the ethical statement has been amended on 20 June 2024 at the request of the corresponding author. Changes have been made to include ‘coordinating and associated centres’ and change ‘NRC (in Reunion Island’s Hospital)’ to ‘NRCs’.

