# Supplementary material for: Molecular epidemiology identifies the expansion of the DENV2 epidemic lineage from the French Caribbean Islands to French Guiana and mainland France, 2023 to 2024
Source: Euro Surveill. 2024 Mar 28;29(13):2400123. doi: 10.2807/1560-7917.ES.2024.29.13.2400123 (PMC10979529; doi:10.2807/1560-7917.ES.2024.29.13.2400123)
Supplement: Supplementary Material [file Supplementary_Materials_2400123.pdf]

This supplementary material is hosted by Eurosurveillance as supporting information alongside the article « Molecular epidemiology identifies the expansion of the DENV2 epidemic lineage from the French Caribbean Islands to French Guiana and mainland France, 2023-2024 », on behalf of the authors, who remain responsible for the accuracy and appropriateness of the content. The same standards for ethics, copyright, attributions and permissions as for the article apply. Supplements are not edited by Eurosurveillance and the journal is not responsible for the maintenance of any links or email addresses provided therein.

Supplementary materials

Sample selection

All sample selection strategies used at the French National Reference Center for arboviruses in mainland France and associated centres in the French Caribbean Islands–Guiana region and in the Indian Ocean region correspond to convenience sampling (details below).

CNR – Mainland France
Samples were referred to the French National Reference Center (CNR) in mainland France (Marseille) by hospital and private laboratories in mainland France for DENV diagnostic or diagnostic confirmation and genotyping. All Dengue 2 positive samples tested at the CNR with a Ct value below 33 with a possible infection origin in the French Caribbean Islands were used for sequencing as part of the arboviral genomic surveillance system implemented by the arbovirus NRC in Mainland France. In addition, the dengue virus sequence for strain “Ae-BV-B4” was determined from mosquito excreta collected from a mosquito survey using the MX method [1] from houses with recent dengue cases in Fort-de-France, Martinique, in October 2023.

CNR – French Guiana

As part of the routine local surveillance system, hospital and private laboratories in French Guiana and French Caribbean Islands referred samples to the associated NRC for the French Caribbean islands - Guiana region for DENV diagnostic or serotyping in 2023. Between July and October 2023, a random selection of Dengue 2 positive samples with a Ct value below 28 detected in French Guiana (n=5 including one imported from Guadeloupe) or received from Martinique (n=4) were used for sequencing as part of the arboviral genomic surveillance system implemented by the arbovirus NRC in French Guiana.

CNR – Réunion
As part of the routine local surveillance system, hospital and private laboratories in Réunion referred samples to the associated CNR for the Indian Ocean region for diagnostic or diagnostic confirmation in 2023. All Dengue 2 positive samples tested at French National Reference Center in Réunion between April and December 2023, with a Ct value below 28, were sequenced as part of the arboviral genomic surveillance system implemented by the arbovirus CNR in Réunion.

Sequencing

CNR – Mainland France
A specific set of primers (supplementary table 4) was used to generate eight overlapping amplicons spanning the entire DENV2 genome with the Superscript IV one step RT-PCR System (ThermoFisher Scientific). PCR mixes (final volume 25µL) contained 3µL of nucleic acid extract, 1,25µL of each primer (10µM), 12.5 µL of 2X Platinum SuperFi RT-PCR Master Mix, 6.5µL of

RNAse free water and 0.5µL of SuperScript IV RT Mix. Amplifications were performed using the following conditions: 10 min at 55°C, 2 min at 98°C, followed by 40 cycles with the three following steps: 10 sec at 98°C, 10 sec at 55°C and 1.45 min at 68°C, and a final step at 68°C for 5 min.
The size of PCR products was controlled by gel electrophoresis. For each sample, an equimolar pool of all amplicons was prepared and purified using Monarch PCR & DNA Cleanup Kit (New England Biolabs). After Qubit quantification using the Qubit® dsDNA HS Assay Kit and Qubit 2.0 fluorometer (Thermo Fisher) amplicons were sonicated (Bioruptor®, Diagenode, Liège, Belgium) into 250pb long fragments. Fragmented DNA was used for library building using the Ion Plus Fragment Library Kit with the AB Library Builder System (Thermo Fisher). To ensure the equimolar pooling of the barcoded samples, a real time PCR quantification step was performed using Ion Library TaqMan™ Quantitation Kit (Thermo Fisher). An emulsion PCR of the pools was performed, followed by loading on 530 chips using the automated Ion Chef instrument (Thermo Fisher), and sequencing using the S5 Ion torrent technology (Thermo Fisher), following manufacturer's instructions.
Read data were analyzed with an in-house Snakemake pipeline [1]. Low quality reads (Q<20) were filtered out and read alignment was achieved using BWA MEM (v0.7.17, [2]) using, as a reference, the best match identified by blasting (magicblast, v1.7.7, [3]) sequencing reads using a database of flavivirus sequences including 42 sequences representative of dengue virus 2 genetic diversity. Consensus sequences were called using the ivar (v1.3.1, [4]) consensus command, and a minimum coverage depth of 50x. Regions with insufficient coverage were masked with N characters.

CNR – French Guiana

Whole genome sequencing was performed on an Oxford Nanopore MinION device using R9.4 flow cells (Oxford Nanopore Technologies, UK). Briefly, 8 µl of RNA was reverse transcribed to complimentary DNA using LunaScript RT SuperMix (Invitrogen) and a serotype-specific multiplex PCR was performed with the Q5 High-Fidelity DNA polymerase (Invitrogen) in three distinct pools, to amplify the entire coding region of DENV-2. Primer schemes were adapted from a CDC protocol (CDC Next Generation Sequencing Protocol for DENV-1-4 using Illumina MiSeq, version December 2022). The resulting 400 bp PCR products were pooled, cleaned using AmpureXP magnetic beads (Beckman Coulter, UK), and quantified using a Qubit dsDNA High Sensitivity assay on a Qubit 3.0 instrument (Thermo Fisher Scientific, USA). The samples were then barcoded using the Ultra II End Repair/dA-Tailing Module (New England Biolabs) and the native barcoding kits NBD104 and NBD196 (Oxford Nanopore Technologies), cleaned with magnetic beads, and pooled equimolarly before ligating the AMII adapters with blunt/TA ligase master mix (New England Biolabs). Sequencing libraries were loaded onto the R9.4 flow cell using the ligation sequencing kit LSK109 (Oxford Nanopore Technologies), and sequencing data were collected overnight. Sequence reads were base-called in high accuracy mode and demultiplexed using the Guppy algorithm v3.6 (Oxford Nanopore Technologies). The consensus genome sequences were produced using the Artic network's bioinformatics pipeline (https://artic.network/ncov-2019/ncov2019-bioinformatics-sop.html), which incorporates primer removal, read alignment to a reference genome (OP941843.1) with minimap2, and the nanopolish algorithm (https://github.com/jts/nanopolish) to improve the consensus sequence. Regions with insufficient coverage were masked with N characters.

CNR – Réunion

Genome sequencing of DENV was directly conducted on viral RNA obtained from positive samples. Briefly, viral RNA extraction from serum and plasma samples was performed using the NucliSENS® easyMAG kit on the eMAG automation system (Biomerieux). Subsequently, reverse transcription was performed with the Lunascript RT supermix kit (New England Biolab, MA, USA). Serotype-specific multiplex PCRs were then carried out in two distinct pools, using the Q5 HS high fidelity 2X master mix (New England Biolabs, MA, USA) with primer schemes described in supplementary table 5. The resulting amplicons were pooled and barcoded using the Rapid Barcoding Kit 96 (SQK-RBK110.96) following the manufacturer's guidelines. The libraries were loaded onto R9.4.1 flow cells (FLO-MIN106D) on the Oxford Nanopore MinION platform. Raw data underwent base-calling using the latest super-accurate configuration, followed by filtering for high-

quality reads (mean Q-score > 10) and demultiplexing through the MinKNOW software package. Bioinformatic generation of consensus sequences was then conducted with the ARTIC network's field bioinformatics pipeline (https://github.com/artic-network/fieldbioinformatics), using a DENV2 genome (Genbank accession: NC_001474.2) as a reference.

Sequence datasets

All publicly available sequences for dengue virus 2 were downloaded from the NCBI Nucleotide database, Genbank (keywords: “dengue virus 2”; database accessed on November 22nd, 2023). We filtered the data by: (i) excluding sequences from laboratory strains (adapted, passaged multiple times, obtained from antiviral or vaccine experiments), (ii) excluding sequences that did not belong to the serotype 2 of dengue virus, (iii) keeping only sequences covering more than 85% of the open reading frame (ORF). The remaining sequences were trimmed to their ORF, aligned using MAFFT (version 7.511, [5]) and inspected manually using the program AliView (version 1.0, [6]). By inferring the phylogenetic relationships between public DENV2 genomes from and a set of reference sequences representative of DENV2 genotypes we identified and selected all those belonging to the Cosmopolitan genotype. Based on this dataset, we generated two distinct alignments. The first one combined the sequences from the French Carribbean islands produced in this study (supplementary table 1), all nearly-complete public genomes from the Cosmopolitan genotype, and a set of 34 sequences representative of the other DENV2 genotypes. The second one includes all sequences listed above and the sequences from French Guiana (5), Réunion island (38), and mainland France (2) generated in this study.

Phylogenetic analysis

For both alignments, we performed a Maximum-likelihood (ML) phylogenetic reconstruction with IQ-Tree (version 1.6.12, [8-9]), using the best-fit model identified by ModelFinder and assessed branch support using an ultrafast bootstrap approximation (UFBoot2) (1000 replicates). The alignment (.fasta) and tree (.nexus) files for this analysis are available at https://github.com/rklitting/D2_BR_FCI_epidemic

To evaluate the timing of emergence of the French Caribbean Islands epidemic clade, we reconstructed time-scaled phylogenies with BEAST (v1.10.5, [10]). We used a subset of 75 sequences from the French Caribbean Islands 2023 selected among the 79 sequences available at the time of starting bayesian inference by performing a root-to-tip analysis and removing sequences whose sampling date was incongruent with their genetic divergence. We used two different substitution models (the HKY substitution model with a gamma-distributed rate variation among sites and no partition into codon positions (HKYG4), or the Shapiro-Rambaut-Drummond-2006 (SRD06)), a uncorrelated lognormal (UCLN) clock model clock, and three different coalescent models (constant, exponential and bayesian skygrid). For the constant and exponential coalescent models, we ran single MCMC chains of 50 million states with the BEAGLE computational library [11]. For the bayesian skygrid coalescent model, we ran single MCMC chains of 100 million states. We used Tracer (v1.7, [12]) for inspecting the convergence and mixing, discarding the first 10 % of steps as burn-in, and ensuring that estimated sampling size (ESS) values associated with estimated parameters were all >200. To identify the best fitted model we performed marginal likelihood estimation using path sampling/ stepping-stone sampling. All xml files for these analyses are available at https://github.com/rklitting/D2_BR_FCI_epidemic.

Supplementary tables

ID	Country	Location	Sampling date	Sampling details	Coverage >50X	Coverage >100X	Accession number	Sequencing technology	Sequencing Center	Case details
18085	FRA	MTQ	2013-07-03	Isolate	99.48		PP320854	Ion Torrent	CNR-mainland	Import into mainland France
21784	FRA	MTQ	2014-01-14	Isolate	99.14		PP320855	Ion Torrent	CNR-mainland	Import into mainland France
53681	FRA	MAF	2019-11-20	Isolate	89.8		PP335481	Ion Torrent	CNR-mainland	Import into mainland France
55289	FRA	GLP	2020-02-20	Isolate	98.75		PP335482	Ion Torrent	CNR-mainland	Import into mainland France
65244	FRA	MTQ	2023-02-14	Human	99.46		PP326743	Ion Torrent	CNR-mainland	Import into mainland France
65364	FRA	MTQ	2023-02-18	Human	97.11		PP326744	Ion Torrent	CNR-mainland	Import into mainland France
65866	FRA	GLP	2023-04-18	Human	99.36		PP326745	Ion Torrent	CNR-mainland	Import into mainland France
65877	FRA	MTQ	2023-04-19	Human	96.56		PP326746	Ion Torrent	CNR-mainland	Import into mainland France
66202	FRA	MTQ	2023-05-11	Human	99.48		PP326747	Ion Torrent	CNR-mainland	Import into mainland France
66353	FRA	GLP	2023-04-04	Human	99.36		PP326748	Ion Torrent	CNR-mainland	Local case
66354	FRA	MTQ	2023-04-18	Human	99.26		PP326749	Ion Torrent	CNR-mainland	Local case
66356	FRA	GLP	2023-04-24	Human	99.46		PP326750	Ion Torrent	CNR-mainland	Local case
66357	FRA	MTQ	2023-04-25	Human	99.44		PP326751	Ion Torrent	CNR-mainland	Local case
66358	FRA	GLP	2023-04-25	Human	99.45		PP326752	Ion Torrent	CNR-mainland	Local case
66361	FRA	MTQ	2023-05-11	Human	99.39		PP326753	Ion Torrent	CNR-mainland	Local case
66362	FRA	MTQ	2023-05-12	Human	87.14		PP335483	Ion Torrent	CNR-mainland	Local case
66363	FRA	GLP	2023-05-12	Human	99.36		PP326754	Ion Torrent	CNR-mainland	Local case
66364	FRA	GLP	2023-05-19	Human	99.47		PP326755	Ion Torrent	CNR-mainland	Import into mainland France
66365	FRA	GLP	2023-05-22	Human	99.36		PP326756	Ion Torrent	CNR-mainland	Local case
66366	FRA	GLP	2023-05-20	Human	99.44		PP326757	Ion Torrent	CNR-mainland	Local case
66367	FRA	MTQ	2023-05-19	Human	99.34		PP326758	Ion Torrent	CNR-mainland	Local case
66368	FRA	GLP	2023-05-23	Human	99.4		PP326759	Ion Torrent	CNR-mainland	Local case
66475	FRA	GLP	2023-06-06	Human	97.13		PP326760	Ion Torrent	CNR-mainland	Import into mainland France
66482	FRA	GLP	2023-06-06	Human	99.4		PP326761	Ion Torrent	CNR-mainland	Local case
66483	FRA	MTQ	2023-06-07	Human	99.48		PP326762	Ion Torrent	CNR-mainland	Local case
66484	FRA	MTQ	2023-06-06	Human	98.45		PP326763	Ion Torrent	CNR-mainland	Local case
66485	FRA	MTQ	2023-06-07	Human	99.45		PP326764	Ion Torrent	CNR-mainland	Import into mainland France
66593	FRA	MTQ	2023-06-09	Human	98.98		PP326765	Ion Torrent	CNR-mainland	Local case
66594	FRA	MTQ	2023-06-13	Human	99.47		PP326766	Ion Torrent	CNR-mainland	Import into mainland France
66595	FRA	GLP	2023-06-12	Human	99.46		PP326767	Ion Torrent	CNR-mainland	Local case
66596	FRA	GLP	2023-06-12	Human	99.36		PP326768	Ion Torrent	CNR-mainland	Local case
66597	FRA	GLP	2023-06-13	Human	99.45		PP326769	Ion Torrent	CNR-mainland	Local case
66897	FRA	GLP	2023-06-21	Human	99.4		PP326770	Ion Torrent	CNR-mainland	Local case
66898	FRA	GLP	2023-06-22	Human	99.43		PP326771	Ion Torrent	CNR-mainland	Local case
66901	FRA	MTQ	2023-06-27	Human	99.45		PP326772	Ion Torrent	CNR-mainland	Local case
66902	FRA	GLP	2023-06-26	Human	99.45		PP326773	Ion Torrent	CNR-mainland	Import into mainland France
66904	FRA	MTQ	2023-06-27	Human	99.46		PP326774	Ion Torrent	CNR-mainland	Local case
66905	FRA	MTQ	2023-06-27	Human	99.47		PP326775	Ion Torrent	CNR-mainland	Local case
66906	FRA	MTQ	2023-06-27	Human	96.05		PP331235	Ion Torrent	CNR-mainland	Local case
66909	FRA	GLP	2023-06-29	Human	99.43		PP326776	Ion Torrent	CNR-mainland	Import into mainland France
67008	FRA	GLP	2023-07-03	Human	99.46		PP326777	Ion Torrent	CNR-mainland	Import into mainland France
67187	FRA	MTQ	2023-07-03	Human	99.46		PP326778	Ion Torrent	CNR-mainland	Import into mainland France
67188	FRA	MTQ	2023-07-03	Human	99.47		PP326779	Ion Torrent	CNR-mainland	Local case

67189	FRA	MTQ	2023-07-05	Human	99.46		PP326780	Ion Torrent	CNR-mainland	Import into mainland France
67190	FRA	MTQ	2023-07-05	Human	99.41		PP326781	Ion Torrent	CNR-mainland	Local case
67192	FRA	GLP	2023-07-06	Human	99.45		PP326782	Ion Torrent	CNR-mainland	Import into mainland France
67198	FRA	MTQ	2023-07-07	Human	99.45		PP326783	Ion Torrent	CNR-mainland	Local case
67201	FRA	MTQ	2023-07-11	Human	99.44		PP326784	Ion Torrent	CNR-mainland	Local case
67561	FRA	MTQ	2023-07-26	Human	99.38		PP326785	Ion Torrent	CNR-mainland	Import into mainland France
67618	FRA	MTQ	2023-08-02	Human	99.45		PP326786	Ion Torrent	CNR-mainland	Import into mainland France
68064	FRA	MTQ	2023-08-09	Human	99.41		PP326787	Ion Torrent	CNR-mainland	Import into mainland France
68252	FRA	GLP	2023-08-23	Human	97.51		PP326788	Ion Torrent	CNR-mainland	Import into mainland France
68628	FRA	MTQ	2023-08-22	Human	96.5		PP335484	Ion Torrent	CNR-mainland	Import into mainland France
68637	FRA	GLP	2023-08-16	Human	99.47		PP326789	Ion Torrent	CNR-mainland	Import into mainland France
68638	FRA	GLP	2023-08-19	Human	99.38		PP326790	Ion Torrent	CNR-mainland	Local case
68639	FRA	GLP	2023-08-17	Human	96.51		PP331236	Ion Torrent	CNR-mainland	Local case
68641	FRA	GLP	2023-08-17	Human	99.68		PP326791	Ion Torrent	CNR-mainland	Import into mainland France
68645	FRA	MTQ	2023-08-21	Human	99.48		PP326792	Ion Torrent	CNR-mainland	Import into mainland France
68649	FRA	MTQ	2023-08-17	Human	98.84		PP326793	Ion Torrent	CNR-mainland	Import into mainland France
68650	FRA	GLP	2023-08-23	Human	99.36		PP326794	Ion Torrent	CNR-mainland	Import into mainland France
68651	FRA	GLP	2023-08-19	Human	95.95		PP326795	Ion Torrent	CNR-mainland	Import into mainland France
68652	FRA	GLP	2023-08-18	Human	99.4		PP326796	Ion Torrent	CNR-mainland	Import into mainland France
68925	FRA	MTQ	2023-08-23	Human	99.41		PP326797	Ion Torrent	CNR-mainland	Local case
68932	FRA	GLP	2023-08-24	Human	99.65		PP326798	Ion Torrent	CNR-mainland	Local case
69057	FRA	MTQ	2023-09-03	Human	99.36		PP326799	Ion Torrent	CNR-mainland	Import into mainland France
69212	FRA	GLP	2023-08-29	Human	97.74		PP326800	Ion Torrent	CNR-mainland	Import into mainland France
69225	FRA	GLP	2023-08-30	Human	99.47		PP326801	Ion Torrent	CNR-mainland	Local case
69227	FRA	MTQ	2023-09-06	Human	99.48		PP326802	Ion Torrent	CNR-mainland	Import into mainland France
69709	FRA	GLP	2023-09-05	Human	99.64		PP326803	Ion Torrent	CNR-mainland	Import into mainland France
69710	FRA	MTQ	2023-09-05	Human	99.47		PP326804	Ion Torrent	CNR-mainland	Local case
69711	FRA	GLP	2023-09-07	Human	99.15		PP326805	Ion Torrent	CNR-mainland	Local case
69712	FRA	GLP	2023-09-07	Human	99.4		PP326806	Ion Torrent	CNR-mainland	Local case
69714	FRA	GLP	2023-09-09	Human	99.69		PP326807	Ion Torrent	CNR-mainland	Local case
69716	FRA	MTQ	2023-09-11	Human	99.69		PP326808	Ion Torrent	CNR-mainland	Local case
69717	FRA	GLP	2023-09-12	Human	99.71		PP326809	Ion Torrent	CNR-mainland	Local case
69718	FRA	MTQ	2023-09-12	Human	99.72		PP326810	Ion Torrent	CNR-mainland	Local case
69947	FRA	GLP	2023-09-18	Human	99.37		PP326811	Ion Torrent	CNR-mainland	Local case
69948	FRA	GLP	2023-09-18	Human	99.34		PP326812	Ion Torrent	CNR-mainland	Local case
70053	FRA	MTQ	2023-09-26	Human	100.0		PP326813	Ion Torrent	CNR-mainland	Import into mainland France
70621	FRA	GLP	2023-10-12	Human	99.39		PP326814	Ion Torrent	CNR-mainland	Import into mainland France
70686	FRA	FCI	2023-10-12	Human	99.34		PP326815	Ion Torrent	CNR-mainland	Import into mainland France
70694	FRA	GLP	2023-08-29	Human	99.37		PP326816	Ion Torrent	CNR-mainland	Import into mainland France
70923	FRA	GLP	2023-10-21	Human	99.29		PP326817	Ion Torrent	CNR-mainland	Import into mainland France
70972	FRA	MTQ	2023-10-24	Human	97.03		PP326818	Ion Torrent	CNR-mainland	Import into mainland France
71023	FRA	BLM	2023-10-05	Human	99.0		PP326819	Ion Torrent	CNR-mainland	Import into mainland France
71024	FRA	BLM	2023-10-06	Human	100.0		PP326820	Ion Torrent	CNR-mainland	Import into mainland France
71027	FRA	BLM	2023-10-05	Human	99.99		PP326821	Ion Torrent	CNR-mainland	Import into mainland France
71050	FRA	MTQ	2023-08-07	Human	100.0		PP326822	Ion Torrent	CNR-mainland	Import into

										mainland France
71056	FRA	MTQ	2023-09-12	Human	100.0		PP326823	Ion Torrent	CNR-mainland	Import into mainland France
71057	FRA	MTQ	2023-08-24	Human	99.99		PP326824	Ion Torrent	CNR-mainland	Import into mainland France
71333	FRA	GLP	2023-11-01	Human	99.35		PP326825	Ion Torrent	CNR-mainland	Import into mainland France
71492	FRA	FCI	2023-10-24	Human	92.95		PP326826	Ion Torrent	CNR-mainland	Import into mainland France
71874	FRA	GLP	2023-11-20	Human	99.32		PP326827	Ion Torrent	CNR-mainland	Import into mainland France
72475	FRA	GLP	2023-12-13	Human	99.3		PP326828	Ion Torrent	CNR-mainland	Import into mainland France
72487	FRA	MTQ	2023-12-14	Human	99.3		PP326829	Ion Torrent	CNR-mainland	Import into mainland France
72527	FRA	MTQ	2023-12-11	Human	99.26		PP326830	Ion Torrent	CNR-mainland	Import into mainland France
72533	FRA	MAF	2023-12-14	Human	99.36		PP335480	Ion Torrent	CNR-mainland	Import into mainland France
72536	FRA	MTQ	2023-12-19	Human	99.34		PP326831	Ion Torrent	CNR-mainland	Import into mainland France
72674	FRA	GLP	2023-12-28	Human	99.34		PP326832	Ion Torrent	CNR-mainland	Import into mainland France
72728	FRA	MTQ	2024-01-03	Human	99.42		PP326833	Ion Torrent	CNR-mainland	Import into mainland France
Ae-BV-B4	FRA	MTQ	2023-10-16	Mosquito excreta	86.75		PP326834	Ion Torrent	CNR-mainland	
CNRG5	FRA	MTQ	2023-07	Human		95.97	PP510622	Nanopore	CNR-French Guiana	
CNRG6	FRA	MTQ	2023-07	Human		99.77	PP510625	Nanopore	CNR-French Guiana	
CNRG7	FRA	MTQ	2023-07	Human		99.68	PP510623	Nanopore	CNR-French Guiana	
CNRG8	FRA	MTQ	2023-07	Human		99.80	PP510618	Nanopore	CNR-French Guiana	
CNRG9	FRA	GLP	2023-09	Human		99.79	PP510621	Nanopore	CNR-French Guiana	Travel to Guadeloupe
S33b01	FRA	MTQ	2023-09-12	Human	NA	97.98	PP082846	Nanopore	CNR-Reunion	Travel to Martinique

Supplementary Table 1. Sequence data from the French Carribean Islands. Territory ISO code 3 correspondence: MTQ: Martinique ; GLP: Guadeloupe ; FCI: French Caribbean Islands ; GUF: French Guiana ; REU: Réunion ; For individuals with no history of travel, infection location corresponds to the place of residence. For individuals with a history of travel to dengue endemic areas within a time frame compatible with an infection abroad, if they were residing in mainland France, the location of the infection was considered to be the travel country/area ; if they were residing in French Guyana or Réunion, travel history is specified as dengue as the infection could have been acquired abroad or locally. For sequences produced using Nanopore sequencing technology, whole genome coverage at 100X (consensus calling depth) is specified in the table, for sequences produced using Ion Torrent sequencing technology, whole genome coverage at 50X (consensus calling depth) is specified in the table.

Substitution model	Tree prior	Median	95%HPD	Log marginal likelihood (PS)	Log marginal likelihood (SS)
HKY+G4	Constant	2022-04-21	[2021-09-30:2022-09-05]	-16384.384183085476	-16384.363326010494
SRD06	Constant	2022-04-17	[2021-09-10;2022-10-03]	-16316.4914719013	-16316.6249937853
HKY+G4	Exponential growth	2022-06-23	[2022-01-22;2022-11-03]	-16370.4532213184	-16370.528652041
SRD06	Exponential growth	2022-07-01	[2022-01-10;2022-10-31]	-16292.2510235727	-16292.3927569479
HKY+G4	Skygrid	2022-09-30	[2022-06-25;2022-12-25]	-16357.859996543775	-16357.969713591445
SRD06	Skygrid	2022-09-30	[2022-06-24;2022-12-26]	-16287.460216563675	-16287.723312043188

Supplementary Table 2. TMRCA estimates from BEAST analyses under different substitution models and coalescent tree priors. Shown for each coalescent tree prior is the

median, with the 95% highest probability distribution of TMRCA in parentheses. Also shown is the log marginal likelihood obtained using path-sampling and stepping-stone sampling for each model/prior combination.

ID	Country	Location	Sampling date	Sampling details	Coverage >50X	Coverage >100X	Accession number	Sequencing technology	Sequencing center	Case details
72638	FRA	GUF	2023-12-31	Human	99.33		PP320851	Nanopore	CNR-mainland	import into mainland France
CNRG1	FRA	GUF	2023-07	Human		99.69	PP510620	Nanopore	CNR-French Guiana	autochthonous
CNRG2	FRA	GUF	2023-08	Human		99.79	PP510619	Nanopore	CNR-French Guiana	Local – no travel information
CNRG3	FRA	GUF	2023-09	Human		99.79	PP510624	Nanopore	CNR-French Guiana	Local – no travel information
CNRG4	FRA	GUF	2023-10	Human		99.7	PP510617	Nanopore	CNR-French Guiana	Local – no travel information
S29b09	FRA	REU	2023-10-04	Human		97.98	PP082866	Nanopore	CNR-Reunion	Travel to Bolivia
S29b10	FRA	REU	2023-10-03	Human		97.98	PP082865	Nanopore	CNR-Reunion	Travel to Bolivia
S30b01	FRA	REU	2023-04-15	Human		97.98	PP082864	Nanopore	CNR-Reunion	autochthonous
S30b05	FRA	REU	2023-04-28	Human		97.98	PP082863	Nanopore	CNR-Reunion	Local – no travel information
S30b06	FRA	REU	2023-04-25	Human		97.98	PP082862	Nanopore	CNR-Reunion	Local – no travel information
S30b07	FRA	REU	2023-04-12	Human		97.98	PP082861	Nanopore	CNR-Reunion	Local – no travel information
S31b01	FRA	REU	2023-05-22	Human		97.98	PP082860	Nanopore	CNR-Reunion	Local – no travel information
S31b02	FRA	REU	2023-05-19	Human		97.98	PP082859	Nanopore	CNR-Reunion	Local – no travel information
S31b03	FRA	REU	2023-05-12	Human		97.98	PP082858	Nanopore	CNR-Reunion	Local – no travel information
S31b04	FRA	REU	2023-05-30	Human		97.98	PP082857	Nanopore	CNR-Reunion	Travel to Tanzania
S31b05	FRA	REU	2023-07-7	Human		97.98	PP082856	Nanopore	CNR-Reunion	autochthonous
S31b06	FRA	REU	2023-06-14	Human		97.98	PP082855	Nanopore	CNR-Reunion	autochthonous
S31b07	FRA	REU	2023-07-25	Human		97.98	PP082854	Nanopore	CNR-Reunion	Local – no travel information
S31b08	FRA	REU	2023-07-09	Human		97.98	PP082853	Nanopore	CNR-Reunion	autochthonous
S31b09	FRA	REU	2023-06-07	Human		97.98	PP082852	Nanopore	CNR-Reunion	Local – no travel information
S31b10	FRA	REU	2023-06-27	Human		97.98	PP082851	Nanopore	CNR-Reunion	Local – no travel information
S32b89	FRA	REU	2023-07-20	Human		97.98	PP082850	Nanopore	CNR-Reunion	Local – no travel information
S32b90	FRA	REU	2023-08-16	Human		97.98	PP082849	Nanopore	CNR-Reunion	Local – no travel information
S32b91	FRA	REU	2023-06-17	Human		97.98	PP082848	Nanopore	CNR-Reunion	Local – no travel information
S32b92	FRA	REU	2023-06-19	Human		97.98	PP082847	Nanopore	CNR-Reunion	Local – no travel information
S33b02	FRA	REU	2023-11-08	Human		97.98	PP082845	Nanopore	CNR-Reunion	Local – no travel information
S33b03	FRA	REU	2023-11-06	Human		94.96	PP082844	Nanopore	CNR-Reunion	Local – no travel information
S33b04	FRA	REU	2023-10-12	Human		97.98	PP082843	Nanopore	CNR-Reunion	Local – no travel information
S33b05	FRA	REU	2023-11-14	Human		97.98	PP082842	Nanopore	CNR-Reunion	Local – no travel information
S33b06	FRA	REU	2023-11-09	Human		97.98	PP082841	Nanopore	CNR-Reunion	Local – no travel information
S33b07	FRA	REU	2023-11-08	Human		97.98	PP082840	Nanopore	CNR-Reunion	Local – no travel information
S33b08	FRA	REU	2023-11-08	Human		97.98	PP082839	Nanopore	CNR-Reunion	Local – no travel information
S33b09	FRA	REU	2023-11-18	Human		97.98	PP082838	Nanopore	CNR-Reunion	Local – no travel information
S33b10	FRA	REU	2023-11-21	Human		97.98	PP082837	Nanopore	CNR-Reunion	Local – no travel information

S33b11	FRA	REU	2023-11-17	Human		97.98	PP082836	Nanopore	CNR-Reunion	Local – no travel information
S35b01	FRA	REU	2023-12-27	Human		97.98	PP082835	Nanopore	CNR-Reunion	Local – no travel information
S35b02	FRA	REU	2023-08-14	Human		97.98	PP082834	Nanopore	CNR-Reunion	Local – no travel information
S35b03	FRA	REU	2023-12-18	Human		97.98	PP082833	Nanopore	CNR-Reunion	Local – no travel information
S35b04	FRA	REU	2023-12-15	Human		97.98	PP082832	Nanopore	CNR-Reunion	Local – no travel information
S35b05	FRA	REU	2023-12-11	Human		97.98	PP082831	Nanopore	CNR-Reunion	Local – no travel information
S35b06	FRA	REU	2023-09-28	Human		97.98	PP082830	Nanopore	CNR-Reunion	Travel to Thaïland
S36b01	FRA	REU	2024-01-09	Human		97.98	PP292049	Nanopore	CNR-Reunion	Local – no travel information
S36b02	FRA	REU	2023-12-11	Human		97.98	PP292050	Nanopore	CNR-Reunion	Local – no travel information
S36b03	FRA	REU	2023-12-18	Human		97.98	PP292051	Nanopore	CNR-Reunion	Local – no travel information
S36b04	FRA	REU	2024-01-02	Human		97.98	PP292052	Nanopore	CNR-Reunion	Local – no travel information
S36b05	FRA	REU	2023-12-15	Human		97.98	PP292053	Nanopore	CNR-Reunion	Local – no travel information
S36b06	FRA	REU	2024-01-03	Human		97.98	PP292054	Nanopore	CNR-Reunion	Local – no travel information
70359	FRA	Limeil-Brevannes	2023-10-05	Human	86.7		PP320853	Ion Torrent	CNR-mainland	autochthonous
68062	FRA	Gardanne	2023-8-10	Human	59.52		PP320852	Ion Torrent	CNR-mainland	autochthonous

Supplementary Table 3. Sequence data from other French territories including French Guiana, Réunion, and Mainland France. Territory ISO code 3 correspondence: MTQ: Martinique ; GLP: Guadeloupe ; FCI: French Caribbean Islands ; GUF: French Guiana ; REU: Réunion ; For individuals living in endemic areas with no history of travel or no travel information, infection location corresponds to the place of residence. For individuals with a history of travel to dengue endemic areas within a time frame compatible with an infection abroad, if they were residing in mainland France, the location of the infection was considered to be the travel country/area ; if they were residing in French Guyana or Réunion, travel history was specified as dengue infection could have been acquired abroad or locally. For sequences produced using Nanopore sequencing technology, coverage at 100X (consensus calling depth) is specified in the table, for sequences produced using Ion Torrent sequencing technology, coverage at 50X (consensus calling depth) is specified in the table.

Forward primers	Sequence (5’ to 3’)	Reverse primers	Sequence (5’ to 3’)
D2_F1	agtwgttagtctacgtgsaccgac	D2_antilles_R1_rc	catwgcacaggtcacratrcc
D2_antilles_F2	ctccatggtagayagaggatg	D2_R2	caacacaaayagagcttggaaytc
D2_F3	atyatgcaggcaggaaaacgatc	D2_antilles_R3_rc	gtgcagctcacyttccatgc
D2_antillesF4	gcagctggactactcttaag	D2_antilles_R4_rc	gagttgagatgtatcctctagcygctat
D2_antilles_F5	gaggacttccrataagataycaaacc	D2_R5_rc	cagtattattgaagctgctatcc
D2_antilles_F6	cgtacaatcaygctcttagtg	D2_R6_rc	gtgaygaytcccctatgtcacac
D2_F7	cctaacaaaaggaggaccaggac	D2_antilles_R7_rc	tctcgatatratgtccattactg
D2_antilles_F8	gtgcaaagaccgacaccaag	D2_R8_rc	agaacctgttgattcaacagcacc

Supplementary Table 4. Amplification primers for whole genome sequencing of DENV2 in the CNR – mainland France.

Name	Sequence	Pool
D2V4-1250_1_LEFT	TCAATATGCTGAAACGCGAGAGA	1
D2V4-1250_1_RIGHT	TCTTCCCCTGAGTGAGGTGTTA	1
D2V4-1250_2_LEFT	ACAGAGGATGGGGAAATGGATG	2
D2V4-1250_2_RIGHT	CCACTGCCACATTTCAGTTCTT	2
D2V4-1250_3_LEFT	AGTGGGGTCTCATGGACTATGA	1
D2V4-1250_3_RIGHT	TCCAGGAACAGTGCCATTCC	1
D2V4-1250_4_LEFT	GAGGTGAGGATGGATGCTGGTA	2
D2V4-1250_4_RIGHT	TCCTTCTTTGTAAACTCCGGCT	2
D2V4-1250_5_LEFT	GCATGGTACCTGTGGGAAGT	1
D2V4-1250_5_RIGHT	TGGCACCCATTTCTGAAATGTC	1
D2V4-1250_6_LEFT	GGGAAGACTGTTTGGTTTGTTCC	2
D2V4-1250_6_RIGHT	AAACCCATCTCGTTTGCCATGG	2
D2V4-1250_7_LEFT	CCACACTGGATAGCAGCTTCAA	1
D2V4-1250_7_RIGHT	TCGTGTCCTGGTCCTCCTTTT	1
D2V4-1250_8_LEFT	TCAAAAGAGGAGAAACGGACCA	2
D2V4-1250_8_RIGHT	CACATGTACCATATGGCTCTGCT	2
D2V4-1250_9_LEFT	GCACGTGAGGCTGTTGAAGA	1
D2V4-1250_9_RIGHT	CCGCACCATTGGTCTTCTCTTT	1
D2V4_1250_10_LEFT	GAAGTCTTACGCCCAAATGTGG	2
D2V4_1250_10_RIGHT	TCTGTGCCTGGAATGATGCT	2

Supplementary Table 5. Amplification primers for whole genome sequencing of DENV2 in the CNR - Réunion.

References

1. L'Ambert G, Gendrot M, Briolant S, Nguyen A, Pages S, Bosio L, Palomo V, Gomez N, Benoit N, Savini H, Pradines B, Durand GA, Leparc-Goffart I, Grard G, Fontaine A. Analysis of trapped mosquito excreta as a noninvasive method to reveal biodiversity and arbovirus circulation. Mol Ecol Resour. 2023 Feb;23(2):410-423.

2. Johannes Köster, Sven Rahmann, Snakemake—a scalable bioinformatics workflow engine, *Bioinformatics*, Volume 28, Issue 19, October 2012, Pages 2520–2522, https://doi.org/10.1093/bioinformatics/bts480

3. arXiv:1303.3997

4. Boratyn GM, Thierry-Mieg J, Thierry-Mieg D, Busby B, Madden TL. (2019) Magic-BLAST, an accurate RNA-seq aligner for long and short reads. BMC Bioinformatics 20: 405.

5. Grubaugh, N.D., Gangavarapu, K., Quick, J. et al. An amplicon-based sequencing framework for accurately measuring intrahost virus diversity using PrimalSeq and iVar. Genome Biol 20, 8 (2019). https://doi.org/10.1186/s13059-018-1618-7

6. Katoh K, Standley DM. MAFFT multiple sequence alignment software version 7: improvements in performance and usability. Mol Biol Evol. 2013 Apr;30(4):772-80. doi: 10.1093/molbev/mst010. Epub 2013 Jan 16.

7. Larsson, A. (2014). AliView: a fast and lightweight alignment viewer and editor for large data sets. Bioinformatics30(22): 3276-3278. http://dx.doi.org/10.1093/bioinformatics/btu531

8. Nguyen LT, Schmidt HA, von Haeseler A, Minh BQ, IQ-TREE: A Fast and Effective Stochastic Algorithm for Estimating Maximum-Likelihood Phylogenies, Molecular Biology and Evolution, Volume 32, Issue 1, January 2015, Pages 268–274

9. Minh BQ, Schmidt HA, Chernomor O, Schrempf D, Woodhams MD, von Haeseler A, et al. IQ-TREE 2: New Models and Efficient Methods for Phylogenetic Inference in the Genomic Era, Molecular Biology and Evolution, Volume 37, Issue 5, May 2020, Pages 1530–1534

10. Suchard MA, Lemey P, Baele G, et al.. Bayesian phylogenetic and phylodynamic data integration using BEAST 1.10. Virus Evol. 2018;4:vey016), doi: 10.1093/ve/vey016

11. Ayres DL, Darling A, Zwickl DJ, et al.. BEAGLE: an application programming interface and high-performance computing library for statistical phylogenetics. Syst Biol. 2012;61:170–173. doi: 10.1093/sysbio/syr100

12. Rambaut A, Drummond AJ, Xie D, Baele G and Suchard MA (2018) Posterior summarisation in Bayesian phylogenetics using Tracer 1.7. *Systematic Biology*. **Syy032**
